# Call to action: recognize and prevent the effects of extreme heat on early childhood development and health

**DOI:** 10.3389/fpubh.2025.1654097

**Published:** 2025-08-25

**Authors:** Vanitha Sampath, Devon Payne-Sturges, Natalie Slopen, Nathaniel Harnett, Alison G. Lee, Kari Nadeau, Nat Kendall Taylor, Lindsey Burghardt

**Affiliations:** ^1^Department of Environmental Health, Harvard T. H. Chan School of Public Health, Boston, MA, United States; ^2^Department of Environmental Health Sciences, School of Public Health, University of Michigan, Ann Arbor, MI, United States; ^3^Department of Social and Behavioral Sciences, Harvard T. H. Chan School of Public Health, Boston, MA, United States; ^4^Division of Depression and Anxiety, McLean Hospital, Belmont, MA, United States; ^5^Department of Psychiatry, Harvard Medical School, Boston, MA, United States; ^6^Division of Pulmonary, Critical Care, and Sleep Medicine, Department of Medicine, Icahn School of Medicine at Mount Sinai, New York, NY, United States; ^7^FrameWorks Institute, Washington, DC, United States; ^8^Center on the Developing Child at Harvard University, Cambridge, MA, United States

**Keywords:** childhood, health, extreme heat, prevention, adaptation, mitigation, heat stress, call to action

## Abstract

The frequency and severity of heat waves are expected to worsen with climate change. Exposure to extreme heat, or prolonged unusually high temperatures, are associated with increased morbidity and mortality. The fetus, infant, and young child are more sensitive to higher temperatures than older children and most adults given that they are rapidly developing. During pregnancy, exposure to extreme heat may result in dehydration, inflammation, and reduced blood flow in the placenta potentially triggering preterm birth and increased rates of stillbirth and low birth weight infants. Young children experience a range of immediate health effects from heat, including disruptions in their sleep and learning, and exacerbations of asthma. Long-term impacts include lower cognitive function, reduced ability to concentrate, and adverse outcomes in mental and behavioral health. It is possible to protect children by taking steps to reduce the potential long-term harm of increasing exposure to extreme heat, such as implementing early warning systems, establishing community cooling centers, and expanding support programs to provide cooling systems to homes. Further, adapting existing infrastructure to withstand increased heat through increasing shade as well as the use of cool pavements or cool/green roofs in early care centers and other places children spend time may be efficient ways of mitigating the developmental effects of extreme heat. Finally, preventing future temperature increases by addressing the root causes behind our rapidly heating planet by decreasing use of fossil fuel and investing in renewable energy sources are ultimately needed to ensure healthy child development.

## Introduction

**Figure 1:**
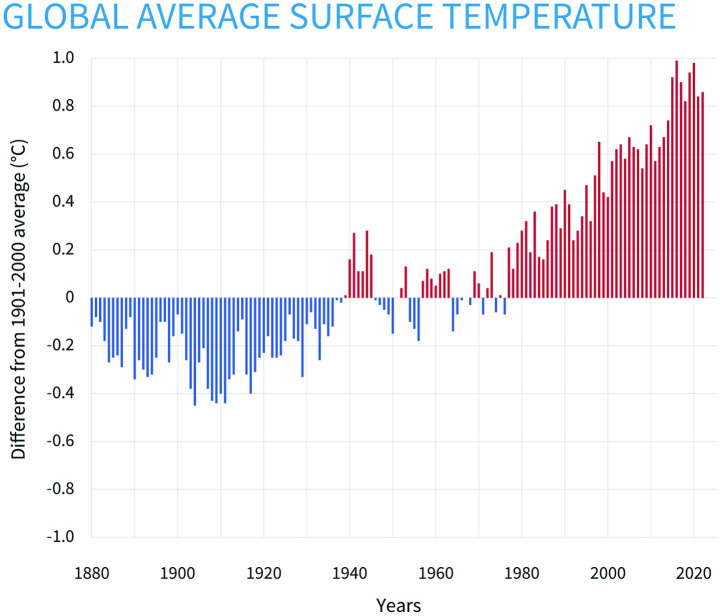
Based on data from the National Centers for Environmental Information, this figure from the National Oceanic and Atmospheric Administration shows yearly surface temperature compared to the 20th-century average from 1880 to 2022. The blue bars indicate cooler-than-average years, and the red bars show warmer-than-average years (62).

Global mean temperatures are rising around the world in part because of human activity (1). Record-setting heat waves are occurring with greater frequency and lasting longer than ever before (2) (Figure 1). The past decade (2015–2024) has been the 10 warmest years on record, with 2024 being the hottest year on record (3). Although policies and global actions have attempted to lower emissions from the burning of fossil fuels to meet the goals of the Paris Accord (limit global temperature rise to 2°C above preindustrial levels by the end of the century and to pursue efforts to hinder temperature increase to below 1.5°C), it is unlikely that this will be met due to inadequate political commitment (4). Exposure to extreme heat is known to put strain on numerous biological systems, which contributes to increased morbidity and mortality, particularly among older individuals and those with heart and lung conditions, and it is thus critical that we take steps to address the potential public health burden of increasing extreme heat.

However, while the health effects of extreme heat are well known, there has been little discussion on the unique impacts of heat on early childhood development. Early development is associated with rapid development across biological systems that support long-term behavioral, physical, and emotional health into adulthood. Indeed, emerging evidence suggests that extreme heat exposure is associated with low birth weight and prematurity, learning loss during the school years, and heat-related illness with life course health implications (5). Given extreme heat puts a strain on key developing systems, understanding the potential impact during early childhood and identifying solutions for ameliorating potential harm, is necessary to advance public health. Here, we offer our perspective and argue for increased attention to, and support for, the impacts of extreme heat on the developing child in the United States. We first highlight extant literature on the biological effects of heat on young children. We further discuss potential solutions that consider the unique developmental needs of children to support them against the adverse effects of extreme heat.

## Effects of heat on babies and young children

Increasing temperatures and heat are likely to impact every region on earth and similarly can affect every organ and cell in the body. Extreme heat refers to when temperatures are significantly higher than normal for any given location. In the brain, extreme heat can lead to slowed cognition and disruptions in emotional functioning—particularly attention, memory, and information processing (6). In the gut, high temperatures can cause the gut lining to become “leaky,” allowing bacteria to penetrate the intestinal barrier and enter the bloodstream (7). During longer periods of exposure, these biological changes can increase the risk of harmful bacteria and toxins reaching the body’s vital organs via the circulatory system. This “invasion,” in turn, further activates the immune system leading to systemic inflammation (8). Heat exposure also increases blood flow to the skin while decreasing its flow to the muscles and other organs, which may cause muscle tissue damage and release proteins into the bloodstream that can cause arrhythmia, kidney damage, and seizures (9). Cells produce heat shock proteins, which act as “chaperones” that stabilize the structure of other proteins that high temperatures could damage. Over short periods of time, heat shock proteins are effective and helpful, but when temperatures are high for too long, they lose their ability to function, and the proteins they protect start to break down (10). This can have a variety of long-term impacts on health, including activation and misdirection of the immune system against proteins that have broken down, leading to increased susceptibility to infections and a decreased response to vaccines (11).

In the prenatal period, heat may result in reduced blood flow in the placenta, dehydration, and inflammation, all of which can trigger preterm birth (12), increase rates of stillbirth (13, 14), or lead to babies born with lower birth weight, all of which are linked to greater risk of a range of poor outcomes later in life including mortality. Additionally, exposure to extreme heat in the first trimester of pregnancy may increase the risk of some birth defects (15). The consequences of being born too early or too small include an increased risk of impaired cognition, reduced growth, and chronic health issues such as cardiovascular disease and diabetes in adulthood (16–22).

During early childhood, biological systems are rapidly developing, making them especially sensitive to exposures from the environment. While heat affects individuals of all ages, the fetus, infant, and young child are more sensitive to heat exposure than most older children and adults, because their smaller bodies heat up more quickly, and they have less capacity to release heat via sweating (23–25). The biological systems that regulate body temperature in infants and young children are less developed and less efficient (26). Infants and young children also rely on adults to seek out cooler environments or get water to drink (24, 25).

## Effects of exposure to extreme heat in infants and children

Exposure to extreme heat in children has been associated with lower cognitive function, reduced ability to concentrate, decreased sleep quality, and adverse outcomes in mental and behavioral health. One analysis of school-age children in the US and Europe calculated that the temperature for optimal concentration is 72°F (22°C) or lower. Student performance on psychological tests and school tasks can be expected to increase on average by 20% if classroom temperatures are lowered from 86°F to 68°F (30–20°C) (27). Conversely, studies show that school performance decreases as temperatures rise. In New York City, for example, learning losses increased by up to 50% when school-day temperatures went above 100°F compared to days above 90°F. In addition, the learning loss from extreme heat events can be lasting: Hotter school days two, three, and even 4 years prior to a test correlate to lower scores (28).

Temperature plays an integral role in sleep quality. During the 2022 heat wave in the UK, researchers studied the impact of high temperatures on infants’ sleep. They found that when temperatures ranged from 96°F to 102°F, infants took longer to fall asleep, had less total sleep, had less efficient sleep, and had more fragmented sleep, and parents’ visits were more frequent during the night. Sleep deficits in infancy increase the likelihood of experiencing emotional and behavioral challenges in early childhood, disrupted language development, and reduced problem-solving skills (29, 30).

The brain detects extreme heat as a threat to wellbeing, which activates the stress response system (31, 32). Excessive activation of the stress response system during pregnancy and in early childhood can disrupt the development of healthy emotional regulation circuits in the developing brain of a child or fetus (33). Excessive heat has been shown to increase violent crime, conflict, and suicide due to a combination of biological and environmental factors (34–36). Experiences of violence are potent activators of the stress response during pregnancy and in young children and can cause long-lasting trauma, decrease a sense of physical and psychological safety and contribute to the development of mental and behavioral health problems in children (37–40).

## Heat and correlated sociocontextual risks

All children face risks from extreme heat, but these risks and their impacts are not evenly distributed and disproportionately affect those in lower socioeconomic groups. The impact of heat is greatest in low-income communities of color (41), where decades of discriminatory zoning and redlining led to the creation of urban heat islands dominated by heat-trapping asphalt, densely concentrated buildings, traffic, industry, and highways (26, 42, 43). Multiple studies show that nearly all US neighborhoods that were subject to redlining are hotter today than non-redlined neighborhoods (42) and have higher levels of air pollution (44). These neighborhoods also tend to have less access to ways of reducing children’s exposure to excessive heat due to systematic underinvestment in infrastructure. For example, lower-income students are more likely to be in schools without adequate air conditioning than higher-income students, and Hispanic and Black households are less likely to have access to air conditioning compared to white households (45). Rural areas are not immune to inequities: More than half of rural US counties have no hospital obstetric services, and the odds of having no local health services for pregnancy and delivery are greatest in lower-income rural counties with more Black women of reproductive age (46). Having to travel long distances to obstetric care for heat-related pregnancy complications likely contributes to higher rates of maternal death, infant death, and childbirth challenges in rural areas (47). This type of documented inequity offers guidance for prioritizing heat-reducing measures where they will have the highest impact on those with the greatest need.

In addition to economic status and geographical location (urban vs. rural), other factors such as diet, living conditions, and stage of development shape the impact that heat has on a child’s health and development. Extreme weather events associated with climate change also impact child health. For examples, wildfires worsen air quality and poorer air quality cause more children to develop asthma and, among children with asthma, exacerbations (48). Extreme heat leads to drought and affects plant and animal health, which impacts the food supply and lowers access to nutritious foods. It also can lead to reduced breast milk production, decreased appetite, and increased burden of infectious diseases such as diarrhea that diminishes an infant’s ability to absorb nutrients, all of which can lead to stunting (49, 50). Therefore, exposure to extreme heat cannot be addressed alone without considering how the conditions in which young children live affect the way that they experience extreme heat.

## Solutions: effective strategies for policy can have multiple positive effects on children’s health

Long-term solutions require us to decrease our dependence on fossil fuels and lower greenhouse gas emissions, which will have a positive effect across multiple domains of children’s health. Practical solutions are being implemented in many parts of the world to mitigate and, separately, adapt to climate change. Mitigation strategies include a wide range of policies and private- sector actions that facilitate a shift from fossil fuels to renewable energy sources, increase energy efficiency and boost natural carbon sequestration. Adaptation strategies include improved access to clean water and food supplies, protection against extreme weather events, availability of cooling technologies, and addressing long-standing inequities that are the root cause of health disparities. Strategies that address high temperatures and other aspects of climate change are also strategies that promote the healthy development of children. Further, studies show that policies that target emissions, for instance, can both benefit children’s health and save on health costs. As just one example, the Regional Greenhouse Gas Initiative—a cooperative effort among 12 states in the northeastern United States to reduce carbon dioxide emissions from power plants—is estimated to have prevented more than 16,000 cases of respiratory illness, 537 new cases of asthma, and other illnesses in children, with substantial savings in health care costs in 5 years (51).

However, there are actions we can take today that can have immediate benefit. Federal agencies must continue to prioritize climate research - to better understand health impacts, to guide policy, and help us be more prepared. A strong Environmental Protection Agency is needed to protect both planetary and human health. A range of practical strategies and approaches to address the effects of excessive heat on children are already demonstrating positive impact in the world (see Table 1). Below we highlight a few examples.

**Table 1:** Organizations and resources addressing climate change risks to children’s health.

Organization	Description	Web site
Children’s Climate risk index, United Nations Children’s Fund (UNICEF)	Global geographical data on the nature and scope of risk to the world’s children from the effects of climate change.	unicef.org/reports/climate-crisis-child-rights-crisis
WHO	World Health Organization report reviews knowledge about the effects of heat waves and makes recommendations for preventive action, including heat health-warning systems, urban planning, and housing design.	who.int./publications/i/item/9789289010948
Intergovernmental Panel on Climate Change: “Climate Change 2023	Summary for Policymakers provides a range of adaptation, mitigation, and near-term actions to reduce the effects of climate change worldwide.	ipcc.ch/srccl/chapter/summary-for-policymakers/
National Center for Medical-Legal Partnerships	Helps medical professionals address legal issues, including documenting the medical necessity of heat-related utility access.	medical-legalpartnership.org/
National Institute of Environmental Health Sciences	Coordinates solutions-focused research to reduce climate change’s health effects and offers educators Climate Change and Human Health Lesson Plans.	https://www.niehs.nih.gov
Smart Surfaces Coalition	Information, tools, and initiatives to help cities incorporate reflective (cool) roofs and pavements, porous pavements, green roofs, and more “to enable cities to thrive despite climate threats, save cities billions of dollars, create jobs, decrease heat, reduce flood risk, slow global warming, and improve city livability, health, and equity.”	smartsurfacescoalition.org
US Climate Resilience Toolkit	Compiles tools, information, case studies, and subject matter expertise from the US federal government to help decision- makers identify local climate threats and vulnerabilities and reduce risks.	toolkit.climate.gov
US Environmental Protection Agency	Climate Change and Children’s Health and WellBeing in the United States” offers a range of information, research, and solutions	epa.gov/cira/climate-change-and-childrens-health-and-well-being-united-states-report
	“Heat Island Cooling Strategies” provides information about how communities: are taking action to reduce excessive heat through vegetation, green and cool roof installation, cool pavements, and smart growth.	https://www.epa.gov/heatislands/heat-island-cooling-strategies
US Global Change Research Program	Collaboration among 14 federal agencies to provide a gateway to authoritative science, tools, and resources to help people and organizations across the country manage risks and respond to changing environmental conditions.	https://www.congress.gov/crs-product/R48478

### Consider where people spend time during pregnancy and childhood

Childcare programs (including informal in-home care settings), preschool programs, K-12 schools, summer and after-school programs, recreational sports, and homes are all places that should be evaluated for their ability to protect people during pregnancy and childhood from exposure to excessive heat and provide what they need to withstand it, such as clean drinking water and shade. Ensuring cooling options are available during pregnancy should be considered integral to prenatal care.

### Improve structural cooling options

The architecture of new buildings, retrofitting of older buildings, and urban planning can be done in a way that reduces heat and makes more efficient use of energy. Many new building materials and power sources, such as “cool pavement” and “cool roofs” with white, reflective, or permeable surfaces, can save money and lives (52). Urban greening campaigns that increase tree canopies and surfaces covered with vegetation can decrease air temperatures and provide shade. Public access to clean drinking water and cooling shelters placed within communities (broadly advertised, including in prenatal clinics, pediatric clinics and hospitals) can better protect people from the effects of extreme heat (53).

### Install air conditioning and other cooling mechanisms

According to the US Environmental Protection Agency, more than $13 billion is lost per year in lower future earnings due to learning losses from school days that are just 7°F higher than current averages. Yet, the annualized cost of installing and maintaining HVAC systems in all US public schools would be less than one-third of that amount (54). Some states used to offer subsidies on air conditioning to low-income residents through the federal Low Income Home Energy Assistance Program (LIHEAP), but this program has recently been dismantled (55, 56). And a range of less-expensive and less power-demanding solutions also exist, from heat sinks (which pump heat underground) to “swamp coolers” (which use evaporation to cool air).

### Provide support for affordable, reliable access to the power grid

Support should be provided for affordable, reliable access to the power grid, with particular emphasis on power from sustainable sources. Ensuring that this support is available during pregnancy and in the postnatal period provides a key opportunity for promoting healthy birth outcomes. During the June 2023 heat wave in Texas, where temperatures reached 118°F, recent investments in solar energy provided up to 15% of the state’s power needs at critical times of the day, preventing wide-scale emergency blackouts (57). Getting an air conditioner is no help if it cannot be powered or if power is unaffordable. In some areas, pediatricians are helping families document the medical necessity of maintaining access to utilities; health insurance may even pay for utility bills in some states (58). Low Income Home Energy Assistance Program (LIHEAP’s) federal Cooling Assistance Program used to help those with low incomes pay energy bills, but the program has recently been shut down (56, 59).

### Develop heat action plans

Communities, policymakers, and healthcare systems can come together to build community resilience. Heat action plans coordinate local government response with other agencies, healthcare facilities, and community organizations (60). For example, health systems can build collaborations to develop local heat response plans that map areas of greatest exposure and incorporate community-derived knowledge about attitudes and practices among the most at-risk members of the community. Community partnerships may identify “heat champions”—respected individuals in the community who can share information about risks and resources during heat waves, particularly ensuring that these alerts reach people during pregnancy and the postnatal period (61).

Drawing upon these examples, we recommend that actions going forward must be addressed at three levels:

- *Protect*: immediate actions such as implementing early warning systems and establishing cooling centers.
- *Adapt*: resources should be allocated toward adapting our services, systems, and infrastructure to be better positioned to withstand increased heat, such as cool pavement, or cool or green roofs, particularly in the spaces where young children spend time.
- *Prevent*: addressing the root causes behind our rapidly heating planet by decreasing the use of fossil fuels and investing in renewable energy sources.

Solutions at each level can be implemented through local, city, state, and federal policy, as well as through direct services to children and families, education, and health care. When possible, solutions should be done in consultation and collaboration with local communities and leaders to address local needs most effectively. Local leadership is key. While efforts to address the root causes of climate change can and should be undertaken at the national and global level, a community’s residents and leaders best know their greatest needs and challenges. Bringing air conditioners to a community without reliable access to the power grid, for example, or planting trees without considering future costs to a community when other forms of shade and cooling may be preferable, are just two examples of well-intentioned but misguided interventions imposed from outside rather than driven by a community.

## Conclusion

The increasing severity and frequency of heat waves will increase the risk of acute and long-term health effects. Fetuses, infants, and young children are more sensitive to the effects of these heat waves as their bodies are still developing and their thermoregulatory capacities are limited. While global temperatures are expected to increase, the extent of these increases will depend on human actions to mitigate greenhouse gases. It is therefore critical that while action is taken via use of early monitoring systems and redesigning our local environments to reduce heat exposure, it is also critical that there is collective action to mitigate global warming by reducing greenhouse gases. Local, state, national, and international agencies must work together to advance meaningful change for the future of children across the globe.

## Data Availability

The original contributions presented in the study are included in the article/supplementary material, further inquiries can be directed to the corresponding author.
